# RASSF1C modulates the expression of a stem cell renewal gene, PIWIL1

**DOI:** 10.1186/1756-0500-5-239

**Published:** 2012-05-16

**Authors:** Mark E Reeves, Melissa L Baldwin, Robert Aragon, Scott Baldwin, Shin-Tai Chen, Xinmin Li, Subburaman Mohan, Yousef G Amaar

**Affiliations:** 1 Surgical Oncology Laboratory, Loma Linda VA Medical Center, 11201 Benton Street (151), Loma Linda, CA 92357, USA; 2 Musculoskeletal Disease Center, Loma Linda VA Medical Center, Loma Linda, CA, USA; 3 Department of Surgery, Loma Linda University School of Medicine, Loma Linda, CA, USA; 4 Department of Pathology, University of California, Los Angeles, CA, USA

**Keywords:** RASSF1C, PIWIL1, Gene expression, RASSF1C target genes, ERK1/2

## Abstract

**Background:**

RASSF1A and RASSF1C are two major isoforms encoded by the Ras association domain family 1 (RASSF1) gene through alternative promoter selection and mRNA splicing. RASSF1A is a well established tumor suppressor gene. Unlike RASSF1A, RASSF1C appears to have growth promoting actions in lung cancer. In this article, we report on the identification of novel RASSF1C target genes in non small cell lung cancer (NSCLC).

**Methods:**

Over-expression and siRNA techniques were used to alter RASSF1C expression in human lung cancer cells, and Affymetrix-microarray study was conducted using NCI-H1299 cells over-expressing RASSF1C to identify RASSF1C target genes.

**Results:**

The microarray study intriguingly shows that RASSF1C modulates the expression of a number of genes that are involved in cancer development, cell growth and proliferation, cell death, and cell cycle. We have validated the expression of some target genes using qRT-PCR. We demonstrate that RASSF1C over-expression increases, and silencing of RASSF1C decreases, the expression of PIWIL1 gene in NSCLC cells using qRT-PCR, immunostaining, and Western blot analysis. We also show that RASSF1C over-expression induces phosphorylation of ERK1/2 in lung cancer cells, and inhibition of the MEK-ERK1/2 pathway suppresses the expression of PIWIL1 gene expression, suggesting that RASSF1C may exert its activities on some target genes such as PIWIL1 through the activation of the MEK-ERK1/2 pathway. Also, PIWIL1 expression is elevated in lung cancer cell lines compared to normal lung epithelial cells.

**Conclusions:**

Taken together, our findings provide significant data to propose a model for investigating the role of RASSF1C/PIWIL1 proteins in initiation and progression of lung cancer.

## Background

Lung cancer is the leading cancer killer of both men and women in the United States [1]. Tumor suppressors and growth promoters play a vital role in lung cancer growth and progression. One such tumor suppressor is RASSF1A, which is inactivated in 80–100% of lung cancer cell lines and tumors [2-5]. In addition to human tumor studies, evidence from *in vitro* over-expression and knockout mouse studies demonstrate clearly that RASSF1A is a tumor suppressor [2,6-11].

RASSF1C is the other major isoform encoded by the RASSF1 gene and it is expressed in the majority of human solid tumors. Some reports suggested that RASSF1C may function as a tumor suppressor in ovarian, prostate, renal cancer cells [10-13]. In contrast, we have recently demonstrated that RASSF1C promotes breast and lung cancer cell proliferation [14,15]. Over-expression of RASSF1C led to increased proliferation of the non small cell lung cancer (NSCLC) cell line NCI-H1299, while silencing of RASSF1C expression led to decreased cell proliferation [14]. Consistent with our findings, others have shown that RASSF1C, but not RASSF1A, over-expression in the human lung cancer cell line A549 results in significant accumulation of the β-catenin oncogene, a key player in the Wnt signaling pathway, leading to increased transcriptional activation and cell proliferation [16]. Previously, we have shown that RASSF1C is a binding partner of insulin-like growth factor binding protein 5 (IGFBP-5), which is a member of the IGF binding protein family that has been shown to be critically important in lung cancer progression [17].

There is growing evidence that RASSF1C and RASSF1A have important and distinct roles in cancer cell proliferation. However, it is certainly possible that the interplay of these two molecules may be critical to determining the eventual growth and progression characteristics of lung cancers. In order to better define the functions of RASSF1C, we used microarray expression analysis to investigate the impact of RASSF1C on gene regulation. We hypothesized that over-expression of RASSF1C might either down-regulate the expression of cell growth inhibiting/pro-apoptotic genes or up-regulate the expression of cell growth promoting/anti-apoptotic genes. In this article, we report on RASSF1C modulation of PIWIL1 gene expression in the NSCLC cells.

## Methods

### Cell culture

The human lung cancer cell lines A549, and NCI-H1299, and the normal lung epithelial cell line CRL-9482, were all obtained from American Type Culture Collection (Manassas, VA). Cell culture was carried out as recommended by ATCC.

### Construction of a tet-inducible expression system that expresses RASSF1C

In order to over-express RASSF1C cDNA in human lung cancer cells in a regulated fashion, we chose to use a doxycycline (dox)-inducible Murine Leukemia Virus based retroviral vector to express RASSF1C that was developed at our institution as previously described [14,18].

NCI-H1299 and A549 lung cancer cells were seeded at 1 × 10^5^ cells/well in 6-well plates. After 24 hr of incubation, the cells were transduced with the MLV-based vectors rtTA-GYT (vector without transgene, designated “backbone”), rtTA-GYT-GFP and rtTA-GYT-HA-RASSF1C with different MOI in 6-well plates, using 2 or 3 serial infection cycles as described [17]. After 1–4 days, cells were treated with up to 1 × 10^−6^ M doxycycline (dox) for 48 hr. Transgene expression was assessed by Western blot analysis using anti-HA antibody. Using cells transduced with the rtTA-GYT-GFP vector, we demonstrated that a 10 fold induction of GFP expression can be achieved with a dox concentration of 1ug/ml (data not shown).

### RNA isolation and RT-PCR analysis

Total RNA from human lung cancer cell lines was isolated from confluent cultures using the Absolutely RNA Microprep Kit (Stratagene, La Jolla, CA). 1 μg of total RNA was used in reverse transcriptase (RT) reactions using the superscript kit (Qiagen, Germantown, MD) and the RT reactions were subsequently used to set up real-time PCR reactions using1 μl of RT as a template. The real-time PCR reactions were set up using Syber green PCR master mix (BioRad, Hercules, CA) and the PCR reactions were run using the Opticon 2 PCR machine (BioRad). The PCR reactions were run using the following protocol: 1. incubate at 95°C for 10 min, 2. incubate at 95°C for 15 sec, 3. incubate for 30 sec, 4. go to line 2 for 39 more cycles, 8. melting curve from 60°C to 95°C, read every 1.0°C, 9. incubate at 10°C forever. The RT-PCR reactions were carried out in triplicate and the fold change was calculated using the 2_T_^−ΔΔC^ method [19]. Cyclophyllin was used as a loading control.

### Western blot analysis

Western blot analysis was carried out using standard procedures with detection on the Odyssey® Infrared System (LI-COR Biosciences, Lincoln, NE). Rabbit polyclonal anti-PIWIL1 (ab12337 Abcam, Cambridge, MA), mouse monoclonal anti-HA (Mon HA.11(16B12) Covance, Berkeley, CA), mouse monoclonal p-ERK (E-4) ( sc-7383, Santa Cruz, Biotechnology, CA), mouse anti-panERK (Cat. 610123, BD Transduction Laboratory), rabbit polyclonal RASSF1C antibody (gift from Dr. Geoffrey Clark, JG Brown Cancer Center, University of Louisville, KY), and goat anti-mouse and donkey anti-rabbit fluorescently labeled secondary antibodies (IRDye^@^680 926–32220, LI-COR Biosciences) were used.

### Microarray analysis

Hybridization of 12 μg of labeled cRNA to an Affymetrix U133 plus 2.0 chip was carried out in triplicate and data analyses were carried out at the UCLA Microarray core facility, Department of Pathology [14]. The control sample is RNA from NCI-H1299 cells stably transduced with MLV-backbone (NCI-BB) and the experimental sample is RNA from NCI-H1299 cells stably transduced with MLV-RASSF1C (NCI-1C). Prior to RNA isolation, NCI-BB and NCI-1C cells were treated with 1 μg/ml doxycycline for 48 hr. Data analysis was performed using dChip [20]. Thresholds for selecting significant genes were set at a relative difference > = 1.5-fold (or/and 2-fold), absolute signal difference > = 50, and p < 0.05. Genes that met all three criteria were considered as significant changes. Comparison results with False Discovery Rate (FDR) < 5% was considered as a valid analysis.

### Primers for RT-PCR validation of selected RASSF1C target genes

Primers used for validation:

CREG forward primer: 5 GTGCCCTATTTCTACCTGAGCC 3

CREG reverse primer 5 AGCATTATGTGAACACAAAGGGG 3

EGR1 forward primer: 5 ATGAAGGAACCCTGTTTCCGT 3

EGR1 reverse primer 5 ATGATGGAGTAGATGGTGGG 3

PIWIL1 forward primer 5 GCAAAAGGTCACAGCAGACA 3

PIWIL1 reverse primer 5 CCTCCCATCTTGCAGTTCAT 3

PTGS2 forward primer 5 ATCACAGGCTTCCATTGACC 3

PTGS2 reverse primer 5 CAGGATACAGCTCCACAGCA 3

Cyclophillin forward primer 5 GCATACAGGTCCTGGCATCT 3

Cyclophillin reverse primer 5 TCTTGCTGGTCTTGCCATTC 3

### Immunostaining

NCI-H1299 lung cancer cell lines stably transduced with either MLV-back bone or MLV-HA-RASSF1C were immunostained for the PIWIL1 protein. Cells were fixed with methanol for 10 min and washed 3 times with PBS. Cells were then incubated with rabbit polyclonal Anti-PIWIL1 (ab12337 Abcam, Cambridge, MA) at 1/1000 dilution for 2 hr. Cells were washed 3 times with PBS and incubated with Alexa Fluor 488 antibody (goat anti-mouse IgG, Invitrogen, Carlsbad, CA) at 1/1000 dilution for 1 hr. Cells were analyzed using a fluorescent microscope with the appropriate filters.

### Silencing of RASSF1C expression in lung cancer cells

Lung cancer cells (NCI-H1299) were plated at 5000/well in 96–well plates 24 hr before infection and cells were infected with Mission® Non-Target shRNA Control Transduction Particles or with multiple Mission® Lentiviral shRNA Transduction Particles (NMID: NM_007182) for silencing RASSF1C (Sigma, St. Louis, MO) as previously described [14]. The NCI-H1299 lung cancer cells used in this study do express RASSF1C but not RASSF1A. Knockdown validation of RASSF1C expression was assessed by Western blot and qRT-PCR using RASSF1C antibody and RASSF1C specific primers*,* respectively.

### Treatment of cells with the MEK/ERK inhibitor, CI-1040

Lung cancer cells were treated with 1 μM CI-1040 (Sigma) for 18 hr, then cells were collected for total RNA and protein lysate preparations for RT-PCR and Western blot analyses, respectively.

## Results

### Establishment of inducible stable NSCLC and normal lung epithelial cell lines

**Figure 1:**
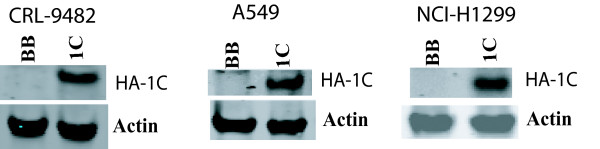
**Western blot analysis of normal lung epithelial cells (CRL-9482) and lung cancer cells (NCI-H1299 and A549) stably transduced with MLV-backbone (BB) or MLV-HA-RASSF1C (1C) treated with 1 μg/ml doxycycline for 48 hrs.** The anti-HA tag antibody detected a HA-RASSF1C fusion protein of the expected size.

NSCLC cell lines (NCI-H1299 and A549) and normal lung epithelial cells (CRL-9482) were infected with retroviral vectors to create stable cell lines that inducibly over-express the gene of interest in the presence of doxycycline. Figure 1 shows that these cell lines stably over-express RASSF1C after treatment with doxycycline.

### Identification of novel RASSF1C target genes in NSCLC cells

We have previously demonstrated that inhibition of RASSF1C expression decreases, and over-expression of RASSF1C increases, breast and lung cancer cell proliferation [14,15]. This suggests that RASSF1C may have an opposite and distinct function from that of RASSF1A in lung cancer. The observed increase in cell proliferation in NCI-H1299 cells over-expressing RASSF1C predicted that over-expression of RASSF1C might either down-regulate the expression of cell growth inhibiting/pro-apoptotic genes or up-regulate the expression of cell growth promoting/anti-apoptotic genes. To answer this question, Affymetrix-microarray analysis was performed using NCI-H1299 cells over-expressing RASSF1C to identify RASSF1C target genes as outlined under Materials and Methods. The microarray datasets show that RASSF1C over-expression modulates the expression of many genes that are involved in cancer development, up-regulating genes involved in cell growth and proliferation, and down-regulating genes involved in cell death (Table 1), suggesting a potential cell growth/proliferation promoting role for RASSF1C in lung cancer cells. The microarray data is consistent with our previous findings that over-expression of RASSF1C resulted in a statistically significant increase in lung cancer cell proliferation [15].

**Table 1:** List of selected novel RASSF1C target genes identified in NSCLC (NCI-H1299) cells

**Cell Cycle and Growth**		
** Gene**	**Gene Description**	**Fold change**
BHLHB2	basic helix-loop-helix domain containing, class B, 2	3.06
CAV1	caveolin 1, caveolae protien, 22 kDa	2.9
CLU	clusterin	3.66
CREG1	cellular repressor of E1A-stimulated genes 1	2.06
EGFR	epidermal growth factor receptor	2.52
EGR1	early growth responds 1	2.35
FGF2	fibroblast growth factor 2 (basic)	2.19
GDF15	growth differentiation factor 15	2.83
HBEGF	herapin-binding EGF-like growth factor	2
KLF4	Kruppel-like factor 4 (gut)	2.36
LIF	leukemia inhibitory factor (cholinergic differentiation factor)	2.14
MDK	midkine (neurite growth-promoting factor 2)	2.51
PLK2	polo-like kinase 2 (Drosophila)	5.79
DAL-1	Differentially expressed in adenocarcinoma of the lung-1 (DAL-1)	-3.32
**Cell proliferation**		
** Gene**	**Gene Description**	**Fold change**
ABCB	ATP-binding cassette, sub-family B (MDR/TAP), member 1	2.12
ALDH1A1	aldehyde dehydrogenase 1 family, member A1	8.07
CAV2	caveolin 2	2.05
COL4A1	collagen, type IV, alpha 1	2.96
COL4A2	collagen, type IV, alpha 2	2.92
CRABP2	cellular retinoic acid binding protein 2	2.18
CRIP2	cysteine-rich protein 2	2.23
EFNB2	ephrin-B2	−2.78
GSN	gelsolin (amyloidosis, Finnish type)	2.51
KISS1	KiSS metastasis-suppressor	−2.97
PIWIL1	piwi-like 1 (Drosophila)	6.87
PLK2	polo-like kinase 2 (Drosophila)	5.79
RASGEF1A	RasGEF domain family, member 1A	3
**Cell death**		
** Gene**	**Gene Description**	**Fold change**
AMIGO2	adhesion molecule with lg-like domain 2	4.16
CARD8	caspase recruitment domain family, member 8	−2.2
CASP4	caspase 4	−1.6
HBEGF	heparin-binding EGF-like growth factor	2
DKK1	dickkopf homolog 1 (Xenopus laevis)	2.62
KLF4	Kruppel-like factor 4 (gut)	2.36
KLF10	Kruppel-like factor 10	2.65

We then confirmed the modulation of selected RASSF1C target genes (CREG1, EGR1, PIWIL1, and PTGS) of interest to us using qRT-PCR (Table 2). We also confirmed that silencing the endogenous expression of RASSF1C, using Lenti-viral siRNA vectors, resulted in the down-regulation of CREG1, EGR1, and PIWIL1 gene expression, and up-regulated the expression of PTGS2 gene (Table 2).

**Table 2:** (A) Selected RASSF1C target genes validated in the NCI-H1299 lung cancer cell line over-expression RASSF1C using RT-PCR. (B) Silencing of endogenous RASSF1C expression had an opposite effect on the expression of RASSF1C target genes

**A**			**B**		
Gene	Fold change	SD	Gene	Fold change	SD
RASSF1C	20	0.05	RASSF1C	−5	0.05
CREG	2.1	0.5	CREG	−2	0.3
EGR1	1.9	0.6	EGR1	−4	0.03
PIWIL1	5.2	0.2	PIWIL1	−2.3	0.04
PTGS	−2.2	0.15	PTGS	1.4	0.11

### RASSF1C modulates the expression of a stem cell renewal gene, PIWIL1

**Figure 2:**
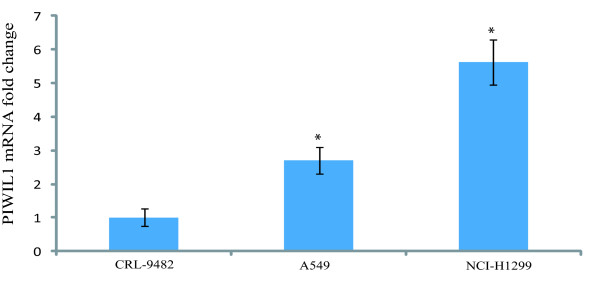
**PIWIL1 mRNA expression in the normal lung epithelial cell line (CRL-9482) and in two NSCLC cell lines (A549 and NCI-H1299) was investigated by RT-PCR analysis. **The RT-PCR analysis shows that the PIWIL1 mRNA levels are significantly higher in the A549 and NCI-H1299 compared to that in CRL-9482. The RT-PCR analysis was carried out using the 2^(−Delta Delta C (T))^ Method.[19].

**Figure 3:**
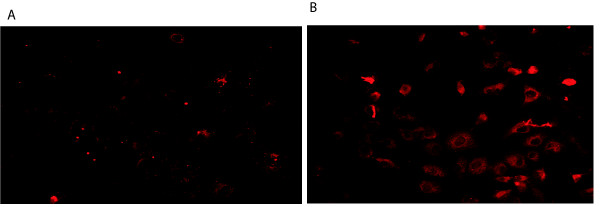
**Immunostaining of PIWIL1 protein in NCI-H1299 lung cancer cell lines stably transduced with either MLV-backbone (NCI-H1299-BB, A) or MLV-HA-RASSF1C (NCI-H1299-1C, B).** Cells were immuno-stained using PIWIL1 antibody as described in the Methods section. The PIWIL1 protein staining is more pronounced in NCI-H1299-1C over-expressing RASSF1C compared to NCI-1299-BB (control) cells clearly demonstrating that RASSF1C over-expression up-regulates PIWIL1 gene expression.

**Figure 4:**
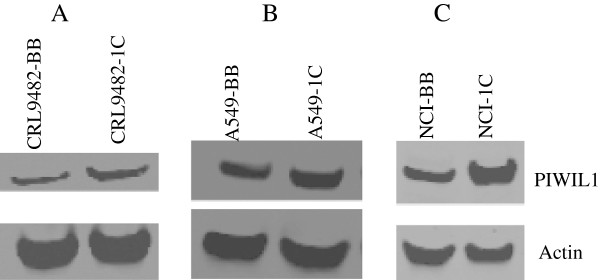
**Western blot analysis of lung primary epithelial cells CRL-9482 (A), A549 (B) lung cancer cells, and NCI-H1299 (C) lung cancer cells stably transduced with MLV-backbone (CRL9482-BB, A549-BB, and NCI-BB) and (MLV-HA-RASSF1C: CRL9482-1C, A549-1C, and NCI-1C) treated with 1 μg/ml doxycycline for 24 hr.** Anti-PIWIL1 antibody detected higher levels of PIWIL1 protein in CRL-9482, A549, NCI-H1299 and cells over-expressing RASSF1C confirming up-regulation of PIWIL1 expression by RASSF1C.

**Figure 5:**
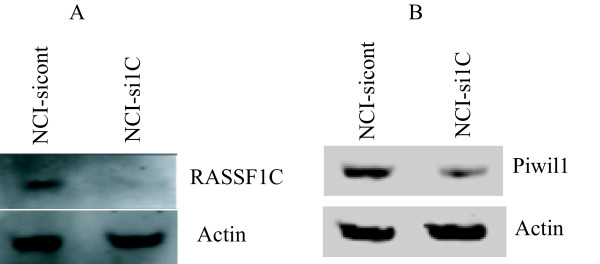
**(A) Western blot analysis of NCI-H1299 lung cancer cells infected with Mission® Lentiviral-shRNA-control particles (NCI-non-target siRNA) and Mission® Lentiviral-shRNA-RASSF1C (NCI-RASSF1C siRNA) to silence endogenous RASSF1C expression.** (B) Anti-PIWIL1 antibody detected less PIWIL1 protein signals in cells with Knocked-down RASSF1C expression compared to control.

PIWIL1 is a stem cell renewal gene that has been implicated in promoting cancer cell growth in a number of different types of cancer [21-24]. However, nothing is known about the role of PIWIL1 in lung cancer cells, making this an interesting gene for further study. We investigated the expression levels of PIWIL1 in lung cancer cell lines and found the PIWIL1 expression is indeed elevated in lung cancer cell lines compared to normal lung epithelial cells (Figure 2). We then further validated the up-regulation of PIWIL1 gene expression (Table 2) in lung cancer cells over-expressing RASSF1C using immunostaining and Western blot analysis (Figures 3 and 4B and C). We also found that over-expression of RASSF1C in primary lung epithelial cells **slightly** up-regulates the expression of PIWIL1 gene expression (Figure 4A). In addition, we show that silencing the endogenous RASSF1C expression down-regulates PIWIL1 gene expression both at the mRNA and protein levels (Table 2 and Figure 5) suggesting that PIWIL1 gene is an authentic target of RASSF1C.

### RASSF1C may activate the MEK-ERK1/2 pathway

**Figure 6:**
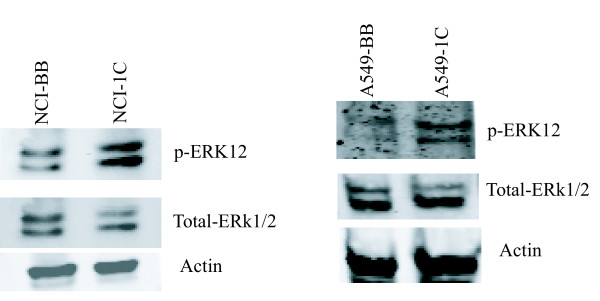
**ERK1/2 activation was assessed in NCI-H1299 and A549 lung cancer cells over-expressing RASSF1C using antibodies that recognize phosphospecific or total ERK1 and ERK2.** The level of phosphorylated ERK1/2 in cells over-expressing RASSF1C (NCI-1C and A549-1C significantly higher compared to control cells (NCI-BB and A549-BB).

**Figure 7:**
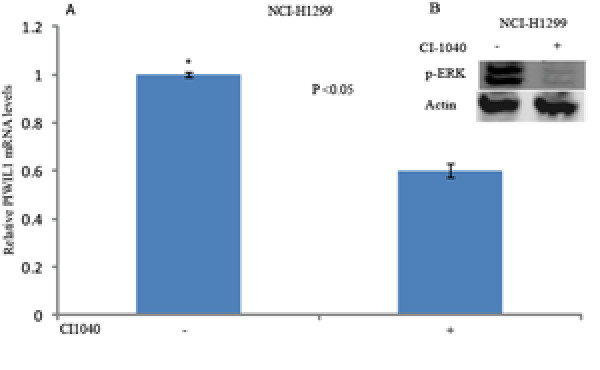
(A) Treatment of NCI-H1299 with the MEK-ERK1/2 pathway inhibitor CI1040 resulted in down-regulating PIWIL1 mRNA levels. (B) Treatment of NCI-H1299 with CI1040 reduces pERK1/2 levels.

In order to identify the pathway(s) that may be activated by RASSF1C in lung cancer, we looked for phosphorylation of ERK1/2, AKT and PI3K. Lung cancer cells over-expressing RASSF1C exhibited increased levels of phosphorylated ERK1/2 compared to control cells (Figure 6), suggesting that RASSF1C may exert its activities on target genes in part through the activation of the MEK-ERK1/2 pathway. To further investigate if RASSF1C actions are mediated by the MEK-ERK1/2 pathway, we treated the cells with the MEK-ERK inhibitor CI-1040 and interestingly found that reduction of the ERK1/2 phosphorylation resulted in the down-regulation of PIWIL1 mRNA levels (Figure 7) in lung cancer cells. Together our findings suggest that RASSF1C actions may be mediated in part by the MEK-ERK pathway.

## Discussion

Previous work in our laboratory has demonstrated that RASSF1C may function to regulate cell proliferation and apoptosis [14,15,17]. We have accumulated substantial evidence suggesting that RASSF1C may promote lung cancer cell growth. Previously, we showed that RASSF1C over-expression increases cell proliferation and siRNA-mediated knockdown of endogenous RASSF1C expression decreases lung cancer cell proliferation, respectively [15]. To begin to address the mechanisms for the effect of RASSF1C on lung cellular growth, we carried out a microarray study to identify novel RASSF1C target genes. The microarray study showed that over-expression of RASSF1C stimulates expression of genes that are associated with cell growth and proliferation and down regulates pro-apoptotic genes (Table 1). Interestingly, some of these target genes have previously been shown to be involved in modulating NSCLC growth and progression. These include the up-regulation of growth promoting genes such as aldehyde dehydrogenase 1A1 (ALDH1A1) [25] and caveolin-1 (CAV1) [26], and down-regulation of growth suppressing genes like the tumor suppressor 4.1B/differentially expressed in adenocarcinoma of the lung-1 (DAL-1) [27]. In addition, expression of Kruppel-like factor 4 (KLF4) [28], Ras guanyl nucleotide exchange factor 1A (RASGEF1A) [29], and polo-like kinase 2 (PLK2) [30] have been reported to exhibit anti-apoptotic and growth-promoting activity in human cancer cells and are also up-regulated by RASSF1C over-expression. We also found that RASSF1C stimulates the expression of other interesting genes such as the stem cell self-renewal gene Piwi-like protein 1 (PIWIL1) [21], and the ABC drug transporter ABCB1 (also known as MDR1) [31]. We have confirmed expression of selected RASSF1C target genes of interest using qRT-PCR in cells that over-express RASSF1C and in cells with knocked-down expression of RASSF1C (Table 2). It should be noted that RASSF1C over-expression had no effect on known RASSF1A gene targets that have been identified in NSCLC cell line A549 and nasopharyngeal carcinoma cell line C666-1 over-expressing RASSF1A [5,32]. This further supports the hypothesis that RASSF1A and RASSF1C have distinct antagonistic functions.

We then further focused on validating the modulation of PIWIL1 gene expression in lung cancer cells over-expressing RASSF1C using immunostaining and Western blot analyses (Figures 3 and 4), we also found that silencing of RASSF1C resulted in reduction of PIWIL1 expression (Table 2 and Figure 5). PIWIL1 protein over-expression has been detected in many tumor types (testicular seminomas, breast, endometrial, gastrointestinal, ovarian, prostate, and soft-tissue sarcoma), but not in normal tissue [21-24]. In this study we also found that PIWIL1 gene expression is significantly elevated in lung cancer cell lines compared to normal lung epithelial cells (Figure 2) underscoring a potential role for PIWIL1 in lung cancer proliferation and progression. Thus, we have chosen PIWIL1 for further investigation for reasons that it may have important functions in cancer stem cell initiation, maintenance, or progression as nothing is known about the function of this interesting gene in lung cancer. We are interested in pursuing studies to determine the mechanism(s) through which RASSF1C modulates PIWIL1 gene expression and its impact on lung cancer cell growth. In this regard, we found that RASSF1C over-expression induces phosphorylation of ERK1/2 in lung cancer cells (Figure 6), raising the hypothesis that RASSF1C could exert its activities on some target genes such as PIWIL1 through the activation of the MEK-ERK1/2 pathway which controls a wide variety of target genes. To further investigate this hypothesis, we inhibited the MEK-ERK pathway using CI-1040, a known inhibitor of the MEK-ERK pathway. We found that inhibition of the MEK-ERK pathway led to the down-regulation of PIWIL1 mRNA levels (Figure 7). Our work is the first to propose a potential link between RASSF1C actions and the MEK-ERK1/2 pathway.

**Figure 8:**
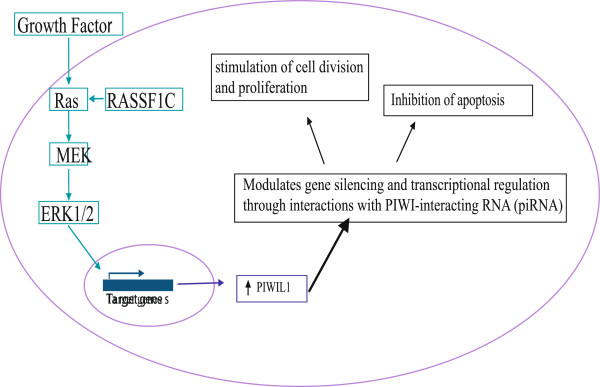
**Hypothetical Model of RASSF1C action in lung cancer cells: we hypothesize that RASSF1C in part activates the MEK-ERK1/2 pathway which controls a wide variety of genes that promote cell division and proliferation.** The model proposes a mechanism through which RASSF1C may impact PIWIL1 expression. Since PIWIL1 is widely over-expressed in tumors compared to normal tissue, it may have important functions in cancer initiation, maintenance, or progression. PIWI-like proteins interact with PIWI-interacting RNA molecules (piRNAs) to form complexes that regulate transcriptional and translational repression leading to inhibition of apoptosis and stimulation of cell division and proliferation. Up-regulation of *PIWIL1*gene expression by RASSF1C is a novel and exciting discovery, suggesting a potential role for RASSF1C in early lung cancer development and progression.

Taken together, our findings provide significant data to propose a model for investigating the role of RASSF1C/MEK-ERK1/2 pathway in initiation and progression of lung cancer cells *in vitro* and *in vivo* (Figure 8). We have shown that RASSF1C modulates PIWIL1 gene expression, potentially through the activation of MEK-ERK1/2 pathway. This leads us to the hypothesis that RASSF1C may play a role in early lung cancer initiation and progression through the activation of ERK1/2 and deregulation of PIWIL1 gene expression in lung cancer stem cells. PIWI-like proteins belong to the argonaute family which is involved in stem cell self-renewal. The PIWI-like proteins do interact with small RNA molecules known as PIWI-interacting RNAs (piRNAs) to form complexes that regulate transcriptional and translational repression leading to inhibition of apoptosis and stimulation of cell division and proliferation [29,30]. Mounting evidence suggests that pathways involved in regulating the self-renewal of stem cells are actually deregulated in cancer stem cells causing uncontrolled expansion of self-renewing cancer cells and tumor formation [33,34].

Our findings are also supported by results from other groups, which have shown that unopposed RASSF1C expression leads to accumulation of the oncogene β-catenin, which is an important part of the Wnt signaling pathway [16]. More intriguingly, elevated levels RASSF1C mRNA are found in all types of lung carcinoma and pancreatic tumors compared to corresponding normal tissue samples further supporting our hypothesis that RASSF1C plays a role in lung cancer development and progression [35,36].

## Conclusion

Identifying RASSF1C target genes related to stem cell self-renewal and survival that include PIWIL1, ALDH1A1, and ABCB1, and linking the activities of RASSF1C to the MEK-ERK pathway are excitingly novel findings that would allow us to pursue future studies to investigate the impact of RASSF1C and the underling pathway(s) associated on lung cancer development and progression.

## Competing interests

The authors declare that they have no competing interests.

## Authors’ contributions

MR participated in the design of the study, contributed to data analysis, and drafting of the manuscript. MB carried out the western blot analysis and apoptosis assays. RA performed RNA and RT-PCR work. SB carried tissue culture and gene cloning work. SC prepared and viral vectors and viral cell transduction. XL carried out microarray hybridization and analysis. SM participated in drafting the manuscript. YA designed and supervised the study, carried out the gene silencing and over-expression work, and contributed to data analysis and drafting of the manuscript. All authors read and approved the final manuscript.
